# Accuracy of positive airway pressure titration through telemonitoring of auto‐adjusting positive airway pressure device connected to a pulse oximetry in patients with obstructive sleep apnea

**DOI:** 10.1111/crj.13658

**Published:** 2023-06-29

**Authors:** Antonio Foresi, Tommaso Vitale, Rosaria Prestigiacomo, Piera Ranieri, Marcello Bosi

**Affiliations:** ^1^ Lung Function and Sleep Unit ASST Nord‐Milano, Sesto San Giovanni Hospital Milan Italy; ^2^ Department of Medicine and Rehabilitation Istituto Clinico Sant'Anna Brescia Italy; ^3^ Sleep Unit Villa Igea, Ospedali Privati Forlì Forlì Italy

**Keywords:** oximetry, sleep apnea, telemedicine, titration

## Abstract

**Purpose:**

In COVID‐19 era, all forms of access of patients to the sleep units should be reduced as much as possible when implementing telemedicine. In the field of obstructive sleep apnea (OSA) therapy with positive airway pressure (PAP) devices, telemedicine includes the use of built‐in software (BIS) and storage of PAPs and remote‐controlled data (BISrc data) that are processed and transmitted daily to sleep units.

We compared two methods of evaluating the final residual severity of OSA patients in home PAP titration: BISrc data versus nocturnal portable multichannel monitoring (PM) data in PAP (reference method) and to verify whether the efficacy PAP therapy guided by BISrc data was clinically adequate.

**Methods:**

We conducted a real‐life prospective study in newly diagnosed patients with OSA. Patients used an auto‐adjusting positive airway pressure (AirSense 10 ResMed) with a pulse oximeter that allows daily transfer of BISrc data (apnea hypopnea index [AHI] and SaO_2_) and remote changes in ventilator setting. Once the PAP titration was completed, the pressure value or ranges were kept constant for 3 days and home PM was repeated.

**Results:**

There were 41 patients with moderate to severe OSA who completed the study. When considering AHI only, the diagnostic accuracy of BISrc on the third day was equal to 97.5%; when considering AHI > 10/h, ODI > 10/h, and SaO_2_ < 90%, the diagnostic accuracy slightly decreased to 90.2%.

**Conclusion:**

In clinical practice, the two measurement methods are equivalent. The use of BISrc data for home titration would reduce the access to sleep units. We urge that widespread use of BISrc be promoted in the current practice of management of OSA.

## Graphical abstract

To verify whether PAP titrations in patients with obstructive sleep apnea through remote‐controlled data (AHI and SaO_2_) from autoPAP with a pulse oximeter are accurate, we compared AHI and SaO_2_ values with data from a standard nocturnal portable multichannel monitoring. In clinical practice, the two measurement methods are equivalent.

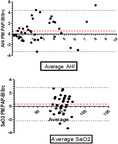

**Abbreviations**
AHI: apnea hypopnea index
ANOVA: analysis of variance
APAP: auto‐adjusting positive airway pressure
BIS: built‐in software
BISrc: built‐in software remote controlled
BMI: body mass index
CI: confidence Interval
CPAP: continuous positive airway pressure
ESS: Epworth Sleepiness Scale
LLN: lower limit of normality
ODI: oxygen desaturation index
OSA: obstructive sleep apnea
PAP: positive airway pressure
PM: portable multichannel monitoring
SaO_2_
: arterial oxygen saturation

## 1 INTRODUCTION

Continuous positive airway pressure (CPAP) is currently the standard treatment for obstructive sleep apnea (OSA). The American Academy of Sleep Medicine (AASM) recommends an attended laboratory procedure to detect optimal CPAP as the gold standard for titration of CPAP to a therapeutic pressure.^1^ However, the alternative method of a home autotitrating machine (auto‐adjusting positive airway pressure [APAP]) has been shown to be equally efficacious.^1^

In the pre‐COVID 19 era, despite an extensive body of literature, there were no exhaustive and consensual statements on how to conduct home autotitration in a standardized way. There has been a small but perceived lack of homogeneity in the procedures due to instrumental resources, personnel resources, and accessibility to centers. A common aspect of home titration with APAP is the analysis and processing of data acquired by the built‐in software (BIS stored data) to identify a single therapeutic CPAP value during one or more accesses to the sleep units.^2^, ^3^, ^4^ This perceived lack of standardization has suggested that the home autotitration procedure should be terminated with a nocturnal polygraphy documenting the true efficacy of the single CPAP value.^5^

The COVID‐19 pandemic has profoundly impacted sleep medicine, resulting in a drastic and generalized reduction of all diagnoses, therapy, and follow‐up activities.^6^ One of most important recommendations is to limit as much as possible of all forms of access of patients to sleep units by implementing telemedicine and the use of BIS remote‐controlled (BISrc) data.^7^, ^8^, ^9^ However, data on virtual sleep units are limited.^10^, ^11^

Thus we tried to further validate telemonitoring for home PAP titration under the guidance of BISrc data only by verifying whether the information on the severity control of the OSA assessed at the end of the home PAP titration from BISrc data was comparable with that provided by a contemporary nocturnal portable multichannel monitoring (PM) during PAP (PM‐PAP) at home used as the reference method and by analyzing the clinical efficacy of a home PAP‐titration procedure guided by BISrc data.

## 2 METHODS

### 2.1 Population and study design

We conducted a real‐life observational prospective cohort study in a group of consecutively recruited and newly diagnosed patients with OSA. All investigations and treatment were done as part of routine clinical practice. All procedures performed in studies involving human participants were in accordance with the ethical standards of the 1964 Declaration of Helsinki and its later amendments or comparable ethical standards. Informed consent was obtained from all individual participants included in the study. Data management was anonymized and used only for scientific purposes.

Participants underwent two nocturnal PMs under baseline conditions and during treatment with PAP. OSA was diagnosed according to the recommendations of the International Classification of Sleep Disorders‐ 3rd edition.^12^ After OSA diagnosis, a standard outpatient session of adaptation and training to the home titration of the pressure device was provided for all patients. This was consistent with clinical practice and involved interface selection, initial acclimatization to APAP, settings to optimize comfort, leak, patient–ventilator synchronization, and use of pulse oximetry.

Home PM Embletta® MPR Sleep System (Embla Systems Inc., Bloomberg, USA) consisted of a snoring sensor, an oronasal thermal sensor, a nasal pressure transducer, a thoracic and abdominal effort sensor (with inductive plethysmograph), a pulse oximetry and a position sensor.

An APAP (AirSense 10 ResMed) set at 6–20 cmH_2_O equipped with a dedicated pulse oximeter (Nonin Xpod, Nonin Medical, Inc, Minneapolis, MN, USA) was used. The PAP machines allowed to automatically process and transfer the BIS data on a daily basis via a central secured data center (cloud) to our sleep laboratory with daily display of the parameters of air leaks, pressure, AHI, mean SaO_2_ and allows remote changes in the ventilator setting (AirView ResMed platform). The possible therapeutic pressure was set after a minimum observation period of five nights in order to minimize the variability caused by sleep stages, changes in sleep position between the nights and also to allow sufficient patient adaptation. The observation period was extended if AHI was still between 5 and 10 events per hour. Patients were not switched to CPAP if they had predominant positional OSA, had REM‐related OSA, or promote the patients comfort. Although patients underwent only polygraphy and not full polysomnography, the presence of significant REM‐related events can be deduced with good approximation on the basis of the presence of clusters of severe desaturations. Comfort during titration is routinely assessed by interviewing the patients. The pressure values were daily checked during titration by technicians and manually changed from night to night (if necessary) when the device was used for ≥4 h, with no or limited losses, to try to obtain an AHI ≤ 10/h, and a mean SaO_2_ > 90%. When set, PAP values were always maintained for a further three nights without changes, in the last of which a home night polygraphy (PM‐PAP) was performed. Oxygen desaturation index (ODI; 3% change in SaO_2_) from built‐in software (BIS stored data) of the three nights was also considered. Although manually recorded, these values were added to BISrc data set. During PM‐PAP, manual scoring of apnea‐hypopnea events were detected (and qualified as obstructive, central, and mixed) by comparing the pressure signals on the mask, oximetry, and the thoracic‐abdominal movement signals.

The analysis on the PM records was manually performed by an expert technician and by a doctor who was a specialist on sleep. AASM 2012 criteria for scoring apnea and hypopneas were used.^13^ Apnea was defined as the cessation of airflow through the nose ≥10 s. Hypopnea was defined as the reduction in airflow ≥30% associated with a decrease in oxygen saturation measured by pulse oximetry ≥3%. A standard sleep questionnaire was used for the evaluation of monitoring time.^14^

Patients were also evaluated with ventilatory function tests: spirometry, flow/volume curve, blood gas analysis at rest while awake in a seated position. Subjective sleepiness was investigated with the Epworth Sleepiness Scale (ESS).

PAP‐failure to control OSA (PM‐PAP reference exam) was detected using two definitions: AHI > 10/h (first definition), at least one of three among AHI > 10/h, ODI > 10/h, and a mean SaO_2_ < 90% (second definition).

The exclusion criteria included current inpatient hospitalization, central sleep apneas, neurological disorders, stimulants or sedatives consumption, alcohol or drug abuse, severe chronic lung disease, severe and unstable cardiovascular or metabolic conditions.

### 2.2 Statistical analysis

Data were presented using descriptive statistics: mean values and standard deviation (±*SD*) for continuous variables and absolute numbers plus percentages for categorical variables. For continuous variables the differences between the groups were analyzed using Mann–Whitney *U* test or the Kruskal–Wallis ANOVA test. For categorical variables, the percentages were calculated and compared using the *χ* ^2^ test. The agreement between PM‐PAP and BISrc data of the third day was evaluated with the intraclass correlation coefficient (ICC) and with the Bland–Altman test. The main analyses of the study were the comparisons between AHI, ODI, and SaO_2_ from BISrc data in the last night (BISrc3) with the corresponding PM‐PAP parameters. All statistical analyzes were conducted with IBM SPSS version 20.0.

## 3 RESULTS

The baseline characteristics of recruited patients are shown in Table 1: 31 males and 10 females; 37 patients (90%) were obese (body mass index [BMI] > 30 kg/m^2^); ESS was >10 in 41% of patients; hypertension was present in 55%; only 5 patients were current smoker whereas 14 patients were past smokers. FEV1/VC was below the lower limit of normality in 4 patients, and obstruction was mild.

**TABLE 1:** Demographic, anthropometric, and functional data (awake) in 41 patients with OSA.

	Mean	Minimum	Maximum	*SD*
Age, years	54.5	32	76	12.6
Height, cm	170.4	140	183	10.2
Weight, kg	101.4	53	150	20.3
BMI, kg/m^2^	35	23	52	6.2
Waist, cm	43.5	37	52	3.3
ESS	8.1	2	15	3.4
TLC, L	5.9	2.5	8.2	1.4
FRC, L	2.9	1.1	3,9	0.7
FEV_1_, L	3,0	1.4	4,8	1,4
TLC, % Pred	94.1	76	117	9.1
FRC, % Pred	87.1	48	115	15.6
FEV_1_, % Pred	90.5	59	133	13.2
FEV_1_/SVC, %	81.9	65.8	98.7	7.4
PaO_2_, mmHg	79.7	58.8	97.1	7.5
PaCO_2_, mmHg	39.2	31.1	48.0	3.1
pH	7.4	7.3	7.4	0.03
HCO_3_‐, mmol/L	24.9	22.3	31.0	1.5

Abbreviations: BMI, body mass index; ESS, Epworth Sleep Scale; HP, hypertension; TLC, total lung capacity; FRC, functional residual capacity; FEV_1_, 1‐s forced expiratory volume; PaO_2_, arterial oxygen partial pressure; PaCO_2_, arterial carbon dioxide partial pressure; HCO3‐, bicarbonate ions.

The respiratory variables obtained with PM at baseline ad during PAP are shown Table 2. At baseline, most patients (38/41) showed a severe OSA (AHI > 30/h) and only 3 patients showed a moderate OSA (AHI > 15/h); 6 patients spent less than 15% of reported sleep time in supine position, and 5 patients slept only in supine position; only 3 patients showed a supine predominant OSA (the supine AHI greater than two times the non‐supine AHI). Observational period in telemonitoring lasted between 5 and 9 days. After PAP titration, 24 patients were on fixed CPAP (mean pressure 13 cm H_2_O, range 11–18 cm H_2_O), and 17 patients were on APAP (pressure range 4–18 cm H_2_O). Time spent in supine at baseline and with CPAP/APAP was 47.9% and 60% of reported sleep time, respectively. During PAP, all PM indices were significantly lower than at baseline (*p* < 0.001).

**TABLE 2:** PM indices at baseline and during PAP (CPAP or APAP).

	Baseline		During PAP	
	Mean	*SD*	Mean	*SD*
AHI, events/h	58.7	20.5	2.6	2.4
Supine time	47.9	29.5	60.1	32.7
AHI supine, events/h	65.2	20.6	38	5.1
Mean SaO_2_, %	90.4	3.3	95.3	1.3
ODI, events/h	58.3	21.2	4.8	4.0
T90, %	30.2	21.4	0.5	2.2
Nadir O_2_,%	69.5	7.6	898	3.2

Abbreviations: AHI, apnea hypopnea index; ODI, oxygen desaturation index; SaO_2_, mean nocturnal saturation; T90, total sleep time spent with SaO_2_ < 90%; Nadir O_2_, nadir oxygen saturation.

BISrc data in the last three nights of observation with PAP are shown in Table 3. There are no statistically significant differences between the mean values of monitoring time, AHI and mean SaO_2_ for the three nights (by Kruskal–Wallis test). The ICC for AHI was 0.86 (95% confidence interval [CI] [0.76, 0.92]; *p* < 0.001), for mean SaO_2_, it was 0.87 (95% CI [0.79, 0.93]; *p* < 0.001). Patients with ODI > 10/h were 19.5% (first night), 12.1% (second night), and 14.6% (third night) (*χ* ^2^ test, *r* = 0.87; *p* = ns). When BISrc data of the third night were compared with the values obtained with PM‐PAP for AHI (2.6/h ± 2.4) and mean SaO_2_ (95.3% ± 1.3), differences were not statistically significant. However, an ODI > 10/h was present in 4/41 patients (9.7%) with PM‐PA vs 6/41 patients (14.6%) with BISrc of the third day (*χ* ^2^ test, *r* = 4.44; *p* = 0.035). The agreement assessed with ICC for AHI was 0.74 (95% CI [0.51, 0.86]; *p* < 0.001), for mean SaO_2_ was 0.69 (95% CI [0.43, 0.83]; *p* < 0.001). The agreement as assessed with ICC for ODI3 (mean values of three nights from storage data—ODI3BIS vs. ODI3PM) was 0.76 (95% CI [0.55, 0.87]; *p* < 0.001).

**TABLE 3:** Mean values ± *SD* of the BISrc data of the last three nights in 41 patients.

	First night	Second night	Third night
Time, h	6.8 ± 1.1	6.6 ± 1.2	6.4 ± 1.0
AHI (events/h)	2.2 ± 1.9	2.3 ± 1.7	2.0 ± 1.9
Mean SaO_2_%	94 ± 1.6	94.7 ± 1.1	95.0 ± 1.3
ODI > 10 events/h	8/41 (19.5%)	5/41 (12.1%)	6/41 (14.6%)

Abbreviations: AHI, apnea hypopnea index; ODI, oxygen desaturation index; SaO_2_, mean nocturnal saturation.

**FIGURE 1:**
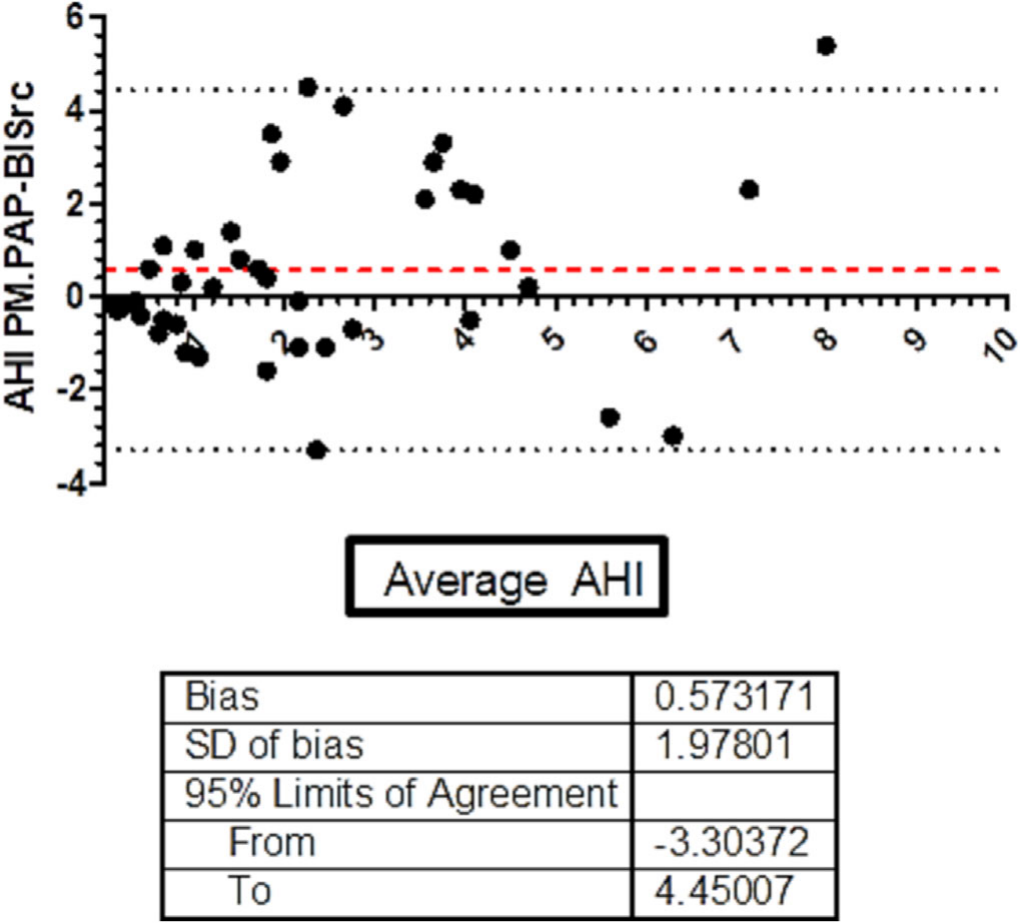
Bland–Altman test between PM‐PAP and BISrc3 data for AHI. The BIAS was 0.57 events/hour with 95% limits of agreement‐3.30 and 4.45. Only 1 patient exceeded the agreement limits. AHI, apnea hypopnea index; BISrc, built‐in software remote controlled; ODI, oxygen desaturation index; PAP, positive airway pressure; PM, portable multichannel monitoring; SaO_2_, mean nocturnal saturation.

**FIGURE 2:**
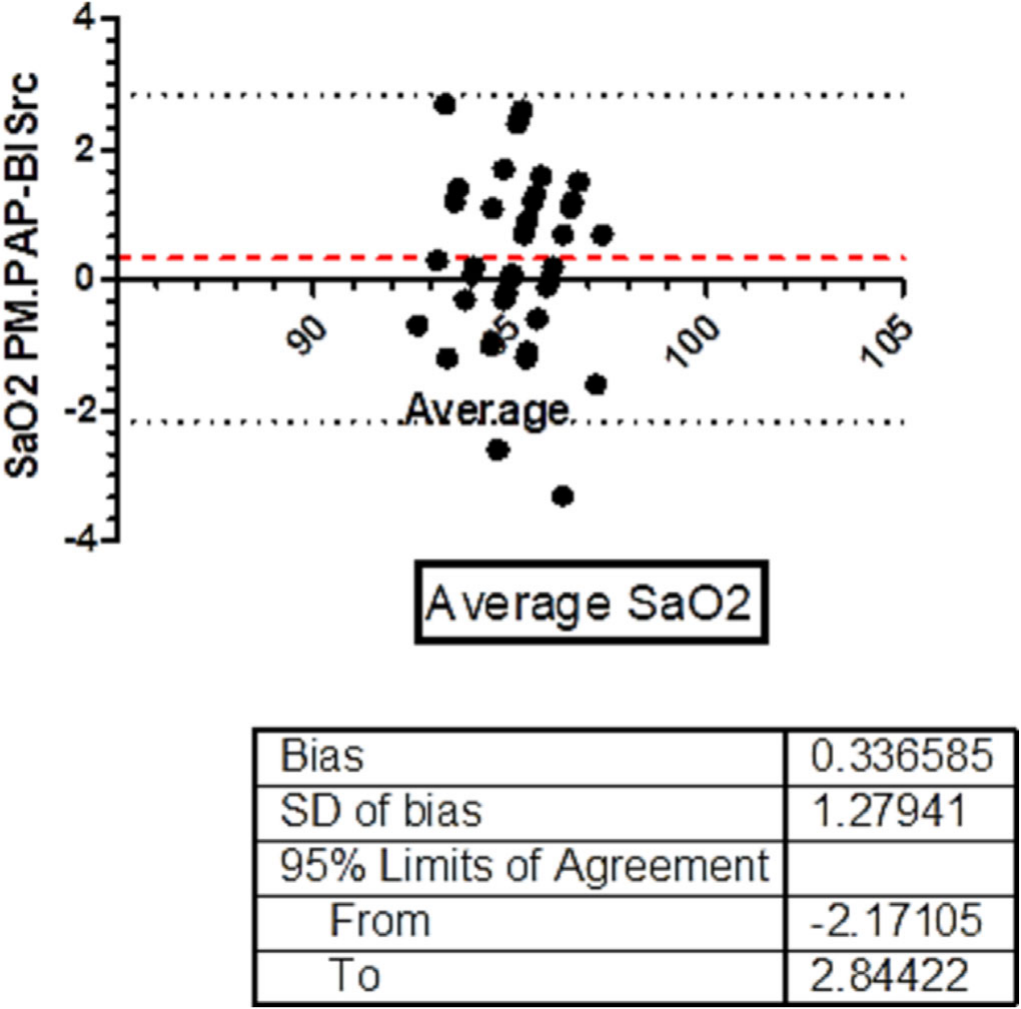
Bland–Altman test between PM‐PAP and BISrc3 data for SaO_2_. The BIAS was 0.33 with 95% limits of agreement −2.17 and 2.84. Only two patients exceeded the agreement limits. AHI, apnea hypopnea index; BISrc, built‐in software remote controlled; ODI, oxygen desaturation index; PAP, positive airway pressure; PM, portable multichannel monitoring; SaO_2_, mean nocturnal saturation.

The analysis of the level of agreement between the two methods (PM‐PAP and BISrc3 data) for AHI and mean SaO_2_ by using the Bland–Altman test is shown in Figures 1 and 2. The AHI‐bias was 0.57/h ± 1.9 (*SD*). Only one patient exceeded the upper limit of agreement. The SaO_2_ bias was 0.33% ± 1.2% (*SD*). Only two patients exceeded the lower limit of agreement. The Bland–Altman found that BISrc3 data slightly underestimate the AHI and SaO_2_. The agreement between ODI3BIS and ODI3PM by Bland–Altman test was ODI3‐bias 0.5% with 95% limits of agreement 6.7 and −7.6. Only two patients exceeded limits of agreement.

A failure to control OSA, that is, an AHI on PM‐PAP > 10/h was present only in 1/41 patients, with an absolute value only slightly above this limit (AHI 10.7/h) and AHI‐BIS rc3 given 0/41 patients using AHI‐BISrc of the third day. The comparison between the two different measurement methods (PM‐PAP data and BISrc3 data) showed an equivalence of the two methods (diagnostic accuracy = 97.5%). The ability of the BISrc data to guide and obtain an effective therapeutic result, with home teletitration occurred in 97.5% of cases.

The analysis using the multiparametric criterion of PAP‐failure (at least one altered between AHI > 10/h, ODI > 10/h, SaO_2_ < 90%) is shown in Table 4. Using PM‐PAP data, patients unsuccessfully treated were 4/41 while patients successfully treated were 37/41. Using BISrc3 data, 35/37 patients were successfully treated whereas 2 patients were correctly classified as undertreated, and the remaining 4 were misclassified (2 false positives and 2 false negatives). The diagnostic accuracy of the BISrc3 data in this case was 90.2% (37/41 patients).

**TABLE 4:** Contingency table analysis using the multiparametric criterion of PAP‐failure (at least 1 altered between AHI > 10 events/h, ODI > 10 events/h, SaO_2_ < 90%) (*χ* ^2^ Pearson's test = 8.1; *p* = 0.004).

Contingency table analysis BISrc third day vs. PM‐PAP				
		PM‐PAP		Total
		Patients +	Patients −	
BISrc third day	Patients +	2	2	4
	Patients −	2	35	37
Total		4	37	41

Abbreviations: AHI, apnea hypopnea index; ODI, oxygen desaturation index; PAP, positive airway pressure; PM, portable multichannel monitoring; SaO_2_, mean nocturnal saturation.

## 4 DISCUSSION

The main finding of the present study is that the information transmitted from the patient's home control of apnea‐hypopneas and of the oximetry parameters detected by the built‐in software of the pressure device was substantially equivalent to that obtainable with a home nocturnal polygraphy. The success rate of home PAP titration conducted with only the use of BISrc data (using both AHI and the multiparametric panel) is very high, making it a robust procedure that can be promoted for clinical use during a pandemic but also in the post‐pandemic period. This can be accomplished in less than 2 weeks. These data are generally conclusive for an interchangeability of the two methods.

Theoretically the goal of PAP is to completely abolish manifestations of OSA.^1^ In reality, residual apneas are commonly present on PAP therapy, and their elimination is not realistically always achievable: The use of AHI with a cut‐off value of<10/h is quite common in the literature and in daily practice, favoring a greater adherence to therapy as well as fewer side effects.^15^

Berry et al.^2^ considered AHI ≤ 12/h, measured at the optimal pressure as an acceptable limit for home PAP titration whereas Rosen et al.^9^ considered a level of AHI < 10 acceptable. Koivumäki et al.^16^ in a home PAP titration study used an ODI cut‐off >10/h and lastly the American Thoracic Society suggested that a follow‐up sleep recording under PAP therapy is not required in patients with a clinical improvement and a device estimation of AHI < 10/h or between 10 and 20/h.^17^

For the multiparametric panel the agreement between the two measurement methods (PM‐PAP data and BISrc3 data) remained very high (90.2%): the fall was due to some patients with AHI < 10/h but oximetric worsening.

Among the first indications for the integrated use of BIS data with oximetry in the titration and follow up of patients using PAP devices were provided by the SomnoNIV‐Group. They stated that recurrent desaturations may reflect upper airway instability, residual obstructive events, decreases in ventilatory response, or repetitive leaks. They found that by integrating clinical data with BIS stored data, it was possible to reduce the number of PM.^18^ Koivumaki et al.^16^ found that almost 80% of patients were adequately treated using a criterion of AHI‐BIS data <5/h whereas the percentage dropped to only 60% by adding also an ODI < 10/h. Thus, the addition of pulse oximetry increased the ability to intercept untreated cases. Oximetry monitoring during home PAP‐autotitration was previously recommended with baseline SpO_2_ < 92% or when BMI ≥ 30 kg/m^2^.

In conclusion, these data define the equivalence between the two measurement methods. The use of BISrc data appears to be adequate for clinical needs and should largely be promoted in place of PM‐PAP especially in this period of COVID‐19 pandemic. Systematically adopting this procedure would eliminate the need for at least 2 face‐to‐face accesses to the sleep units for assembly and disassembly of the polygraph. Our results strongly suggest that identification of pressure should not only preferably be performed by telemedicine, but once the best operating pressure has been identified, should not be confirmed by performing a final PM.

The second result is that home PAP titration conducted under the guidance of telemetric data only was found to be effective and adequate for clinical needs in almost all patients both using only AHI and using multiple parameters. The addition of oximetry data is of value, and it is hoped that PAP devices will be provided with a different algorithm or data transfer that could allow a more detailed analysis of saturation trace. By adopting this procedure, patient's access to sleep units to extract and evaluate the stored BIS data and set the therapeutic pressure would be cancelled.

The AASM recommended considering home autotitrating PAP as equivalent to laboratory titration in reducing the severity of OSA.^1^ The task force had performed a meta‐analysis of three RCTs reporting on the residual AHI: No clinically significant difference in residual OSA severity comparing home autotitrating based on BIS stored data versus in‐laboratory titration was observed. Indeed, an excess of residual events during both PAP titration and follow‐up has been frequently described. Fanfulla et al.^18^ reported that in 300 OSA patients already on home PAP therapy (based on laboratory titration or home autotitration followed by polygraphy), 18.7% of patients still had an AHI_PSG_ > 5/h at follow‐up. This percentage decreased to 8.6% and 4.6%, considering a cut‐off value of 10/h or 15/h. Additionally, Rosen et al.^19^ found that 15% of laboratory titrated and 14% of home autotitrated patients (with an AHI < 10/h as a therapeutic target) had BIS‐stored follow‐up data >10/h. Finally, in more than 12 000 patients on CPAP,^20^ 22.1% had an AHI > 5/h on follow‐up BIS data. Factors associated with residual AHI > 5/h were male sex, age, sedentary lifestyle, OSA severity, cardiovascular comorbidities, and orofacial mask.

In general, there is a growing convergence of the literature in postponing and rescheduling all diagnostic and therapeutic procedures involving access to the sleep unit, in promoting home diagnosis and home autotitration, in particular in promoting the use of telemedicine as much as possible for the entire diagnosis and titration procedure of the CPAP and follow up of the OSA.^7^, ^8^, ^9^, ^21^, ^22^, ^23^, ^24^

A comprehensive and concise overview of what telemedicine is in the diagnosis, treatment, and follow‐up of OSA and the extreme complexity of building a virtual sleep clinic (in terms of technology, structures, human resources, costs, and ethical and legal problems) has been recently outlined by Bruyneel.^23^ Although the literature on home autotitration using BIS stored data is extensive and consolidated, the one with BISrc data to date is still limited.^19^, ^20^, ^21^ Unlike the BIS‐stored data, the BISrc data have the limitation of not allowing manual analysis of the high‐definition curves of air flow, leaks, pressure delivered in the airways, and oximetry; and the parameters transmitted are only those processed automatically by the machine algorithm. Compared with the PAP titration procedure with BIS stored data, the one obtained with remote data transmission, however, cancels face‐to‐face accesses to the sleep center for data processing and the setting of fixed CPAP/APAP values, an important aspect in the current COVID‐19 pandemic.

Finally, the BISrc3 data did not differ from those of the previous two nights in terms of average values. Statistical analysis showed a satisfactory agreement for both AHI and SaO_2_ data detected by the algorithm of CPAP/APAP. Taken as a whole, these data suggest a short‐term internal stability that is not absolute but high and adequate to the pandemic situation but useful also in post‐pandemic times because it limits the need for home sleep studies to confirm the adequacy of the pressure obtained by autotitration.

The study has some limitations: (1) It included a significant although limited, number of patients; (2) most patients had OSA of severe degree; (3) the BISrc data ODI were automatically calculated by the device algorithm; (4) results may be limited to the technology used and marketed by a single manufacturer (ResMed) and possibly applies to the specific device (AirSense 10 ResMed) that has been use. Whether these results apply also to other APAP devices need to be tested.

## 5 CONCLUSIONS

The BISrc data, which also include oximetry, provided information on the severity control of OSA at the end of titration comparable with that provided by a home PM‐PAP. Our real‐life data are conclusive for an interchangeability of the two methods and cast doubt on the necessity of a mandatory final polygraphy for PAP titration. At‐home PAP titration under the guidance of the BISrc data were adequate to the clinical needs and should largely be promoted. Telemonitoring should include oximetry data, and BISrc should allow a more detailed analysis of saturation profile. By systematically adopting this procedure, the need for face‐to‐face access to the sleep unit would be significantly reduced.

## AUTHOR CONTRIBUTIONS

Antonio Foresi was responsible for the study concept, study design, interpretation of results, preparation of the first draft of the manuscript, editing of manuscript drafts, and approval of the final version of the manuscript. Vitale Tommaso was responsible for the data collection and approval of the final version of the manuscript. Rosaria Prestigiacomo was responsible for the data collection and approval of the final version of the manuscript. Piera Ranieri was responsible for interpretation of results, the editing of manuscript drafts, and approval of the final version of the manuscript. Marcello Bosi was responsible for the analysis of data, editing of manuscript drafts, and approval of the final version of the manuscript.

## CONFLICT OF INTEREST STATEMENT

The authors declare no conflict of interest.

## ETHICS STATEMENT

All procedures performed in studies involving human participants were in accordance with ethical standards of the 1964 Declaration of Helsinki and its later amendments or comparable ethical standards. Informed consent was obtained from all participants included in the study. Data management was anonymized and used only for scientific purposes.

## Data Availability

The data that support the findings of this study are available from the corresponding author upon reasonable request.
